# Primary malignant melanoma of the male urethra: Case report and review of literature

**DOI:** 10.1016/j.ijscr.2023.108697

**Published:** 2023-08-19

**Authors:** Quang Nguyen, Huu Thao Nguyen, Xuan Truong Bui, Van Quang Bui, Tien Dung Nguyen

**Affiliations:** a Center for Andrology and Sexual Medicine, Viet Duc University Hospital, Hanoi, Viet Nam; b University of Medicine and Pharmacy, Vietnam National University, Hanoi, Viet Nam; c Department of Surgery, Hanoi Medical Univerity, Hanoi, Viet Nam

**Keywords:** Melanoma, Urethral melanoma, Partial penectomy, Endoscopic inguinal lymphadenectomy, Case report

## Abstract

**Introduction:**

We report a rare case of primary malignant melanoma with inguinal lymph node metastasis in the male urethra.

**Case presentation:**

A 57-year-old male patient presented with a small tumor on the ventral surface of the penis, which was discovered 5 months ago and did not cause pain or discomfort. In the past month, the patient has developed symptoms of urinary incontinence. MRI and PET/CT scans revealed a primary tumor in the penile urethra, but no metastases were found. The patient underwent partial penectomy surgery and laparoscopic bilateral inguinal lymphadenectomy within one month. The pathological combined with immunohistochemical staining confirmed primary malignant melanoma in the urethra with right inguinal lymph node metastasis. Despite complying with surgical and immunotherapy treatment with Pembrolizumab for 18 cycles, the patient was diagnosed with recurrent cancer in the penile stump after 05 months and he passed away after 18 months.

**Discussion:**

Urethral melanoma is a rare and highly invasive type of cancer. It was often diagnosed at a late stage because the initial symptoms were not obvious in the lower urinary tract. Additionally, cancer progressed very quickly, making it difficult to treat.

**Conclusion:**

Urethral melanoma, if detected at a late stage with lymph node metastasis, has a significantly poor prognosis irrespective of the treatment method employed. However, to our best knowledge, very few publications can be found on this disease, and the strategic treatment remained unknown.

## Highlights

• The initial PET/CT scan did not reveal any metastases in the lymph nodes. However, after undergoing endoscopic inguinal lymphadenectomy surgery, we found lymph node metastasis.
• Despite strict and aggressive adherence to surgery (partial penectomy and endoscopic inguinal lymphadenectomy) and immunotherapy with Pembrolizumab, the disease seemed intractable and recurred after 5 months, death after 18 months.
• Case report of primary malignant melanoma of the male urethra, the first of its kind in Vietnam in English literature.

## 1 Introduction

Primary malignant melanoma is primarily located in the skin and eyes, with very few cases occurring in the urethra, accounting for only 0.2 %–1 % of all melanoma cases. The cause is believed to be the development of the urethra from endodermal origin, but melanocytes originate from neuroectodermal tissue during embryonic development [1,2]. According to a systematic analysis conducted by Safadi A et al. in 2014, the average age for detecting cancer is 60 years or older. The prevalence of this type of cancer is three times higher in women than in men. Women are also more prone to early metastasis than men, and this can occur through superficial vulvar and vaginal lymph nodes, inguinal lymph nodes, or distant hematogenous metastases [2]. Urethral melanoma carries a more unfavorable prognosis than cutaneous melanoma. The 5-year survival rate for patients with urethral melanoma is estimated to be 18 % to 20 %, and this rate further declines as the disease advances. Prognosis is particularly grim for patients who exhibit ulceration, a Breslow depth exceeding 3.5 mm, a tumor diameter greater than 15 mm, as well as concurrent nodal or distant metastasis at the time of diagnosis [3,4]. Male urethral malignancies are rare, but they typically occur in the distal urethra near the urethral orifice. Consequently, common symptoms include hematuria, a palpable mass, and signs of obstruction. Obstruction of the urinary tract can manifest as a weak urine stream, dysuria, urinary incontinence, or the inability to control urination [2,5]. For educational and clinical purposes, we present a case report of a 57-year-old male diagnosed with primary malignant melanoma in the penile urethra, with accompanying right inguinal lymph node metastasis. In this report, we share our preliminary diagnostic and treatment experience with this particular case. This paperwork has been reported in line with the SCARE criteria and guidelines [6].

## 2 Case presentation

### 2.1 Patient information

A 57-year-old male patient, who works as a hotel manager and resides in Hanoi, Vietnam, has a good economic status.

### 2.2 Clinical findings

The chief complaint before admission was a small tumor on the ventral surface of the penis, which had not caused any pain or discomfort for 5 months. However, in the past month, he started experiencing symptoms of urinary incontinence after each urination. The patient and his family had no previous medical problems.

The clinical symptoms included a conscious patient with an average physical condition and a BMI of 23, no fever, no weight loss, and no abnormal color changes in the penis and foreskin. The clinical examination revealed a hard and firm tumor measuring 2 cm on the ventral surface of the penis, which corresponds to the urethral position. Additionally, there was a palpable right inguinal lymph node measuring 2 cm.

### 2.3 Diagnostic assessment

**Fig. 1:**
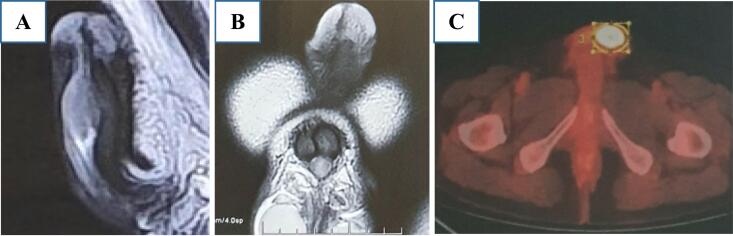
Shows MRI scans (A) and (B) indicating that cancer has invaded the left corpus cavernosum and occupies nearly the entire urethra, (C) A malignant hypermetabolic tumor is observed on the tip of the penis on PET/CT.

The diagnosis was made based on the MRI images. An observed penile cancer mass, measuring 30x25x16mm, with unclear margins and infiltrating the subcutaneous tissue of the foreskin and left corpus cavernosum. A metastatic work-up was requested. The bilateral inguinal lymph nodes showed no abnormal metabolic activity and demonstrated no evidence of metastatic disease (Fig. 1). To prevent further damage, we decided not to conduct the biopsy before surgery.

### 2.4 Therapeutic methods

**Fig. 2:**
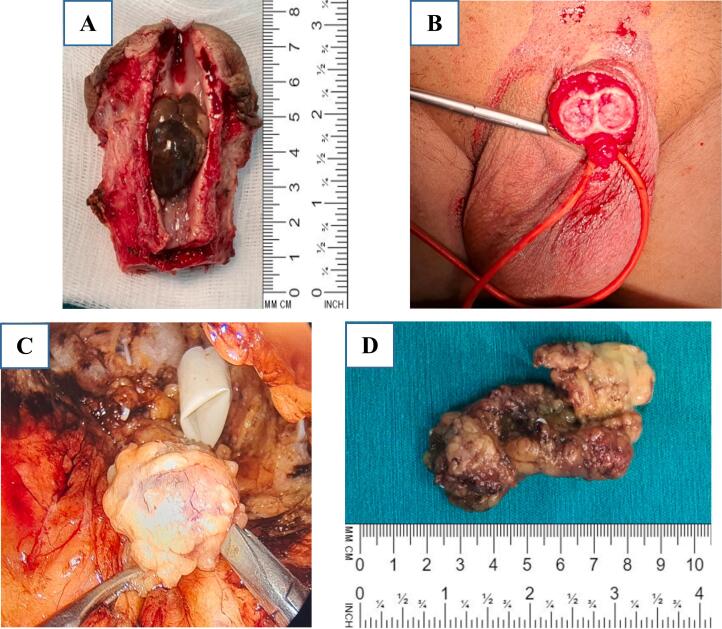
Shows the partial penectomy surgery, which includes the removal of the tumor and laparoscopic bilateral inguinal lymphadenectomy. (A) A longitudinal cross-section of the penis and urethra reveals a tumor mass that measures 2.5 × 2 cm, occupying nearly the entire urethra. (B) The remaining portion of the penis is displayed. (C) An endoscopic image displays lymph node dissection on the right side. (D) An image of the excised chain of potentially malignant lymph nodes is shown.

For local and symptom control, the patient subsequently underwent partial penectomy on March 25, 2021 (Fig. 2A and B), and immediate biopsy results indicated that the melanoma had infiltrated the urethra and the left cavernous and no malignant cells were detected in the rest of the urethra or shaft of the penis.

**Fig. 3:**
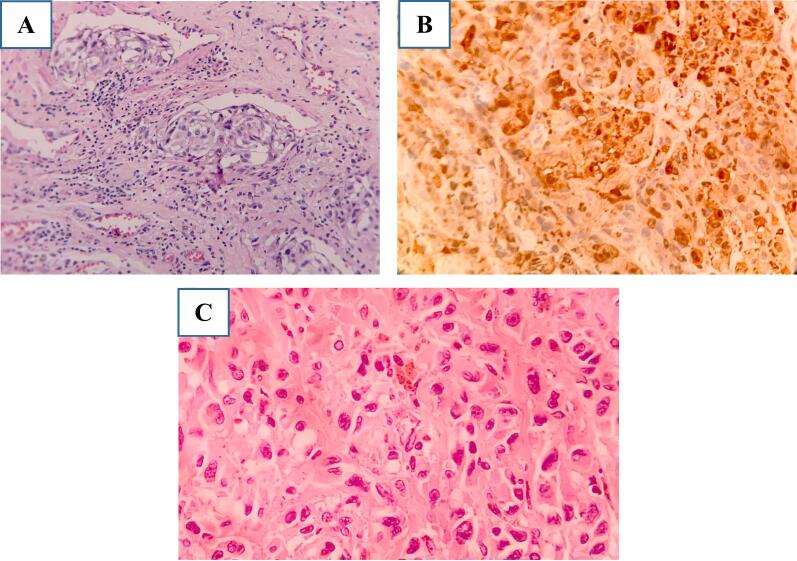
Shows histologic specimens. (A) The HE staining shows the existence of tumor cells with a wide cytoplasm containing melanin pigment. (B) Sections of the main tumor mass show strong positive staining for HMB-45, S-100 protein, Ki67 (15 %), and Melan A. (C) The HE-stained image of metastatic tumor tissue in the right thigh lymph nodes.

To identify the latent metastases, we performed bilateral lymphadenectomy by endoscopic surgery on April 23, 2021. The number of lymph nodes excised from the left was 11, consisting of chronic inflammatory nodes, and from the right were 15 lymphatic nodes with 01 malignant metastatic nodes (Fig. 2C and D). Routine hematoxylin and eosin and immunohistochemical staining (Fig. 3) showed a subepithelial high-grade malignant neoplasm, consistent with melanoma, and revealed melanoma metastasis in the right inguinal lymph node. Genetic testing showed that the patient had 36 gene mutations, with 11 of them being BRAF (exon 11 and 15), and 25 of them NRAS (exon 2, 3, 4). Additionally, PD-L1 was positive at 7 %.

Based on these findings, the patient was diagnosed with primary urethral melanoma, Stage T3N1M0. The patient was started on adjuvant immunotherapy with 18 consecutive doses of Pembrolizumab (Keytruda) 100 mg/dose, two months after the first surgery, and experienced no major side effects.

### 2.5 Outcomes

**Fig. 4:**
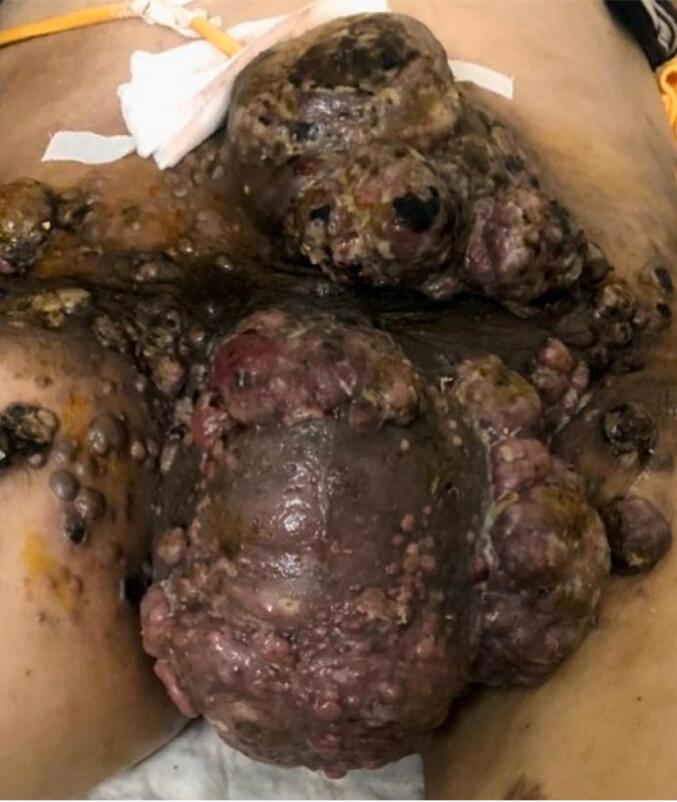
Depicts melanoma that has spread to the skin around the scrotum and pelvis, taken in August 2022.

After two surgeries, the patient did not experience any complications. However, five months after undergoing partial penectomy surgery, a recurrence at the site of the original penis tumor was diagnosed. A third surgery was planned after one week to remove the recurrent tumor. However, during the surgery, it was discovered that the cancer had metastasized to the bladder. Consequently, the penis stump was closed, and a suprapubic cystostomy was recommended. Following the surgery, the patient experienced adverse and unforeseen occurrences, which encompassed swelling and pain at the penis stump. To alleviate the pain in that area, the patient underwent 10 sessions of radiation therapy. In January 2022, metastatic tumors were detected on the skin of the scrotum and pelvic bones (Fig. 4). Two months later, in May 2022, the cancer metastasized to the lungs, and by August 2022, it had spread to the brain. The patient passed away in September 2022 after 18 months of strict adherence and intensive treatment, which included three surgeries, 18 doses of immunotherapy, and 10 rounds of adjuvant radiotherapy.

## 3 Discussion

Primary melanoma of the male urethra is an extremely rare malignant tumor with a high mortality rate. In 2014, in a study conducted by Papeš D et al., 129 articles reporting on 220 patients were searched in PubMed. The median age of the patients was 65 years and the median survival rate was 28 months, with a 5-year survival rate of approximately 10 % for stage A and invasion depth less than 3–3.5 mm [7]. In a retrospective epidemiological study by Sanchez et al. from 1973 to 2010, 1586 cases of malignant primary urogenital tract melanoma, including the kidney, ureter, bladder, and genital organs, were observed. The cases included 1463 female and 123 male patients who were diagnosed through histopathology. The median age of these cases was 66.1 years, and only 11 males had primary melanoma of the urethra [8].

Symptoms of male urethral melanoma often depend on the location of the tumor, and patients usually do not have symptoms in the early stages. However, as the disease progresses, symptoms such as palpable protrusions, urinary obstruction, blood in the urine, abnormal urethral discharge, and sometimes urethral fistula may occur. More than 50 % of patients may have palpable lymph nodes during an examination, which are signs of advanced disease stages and metastasis, and are thus associated with a poor prognosis [2,8,9]. There are currently no screening or early diagnostic tools available, and diagnosis in males relies primarily on inquiry and clinical examination of the penis, foreskin, scrotum, and perianal area for any signs of ulcers, nodules, or other abnormal images. Unfortunately, most symptoms in the male urinary tract are similar to those of chronic prostatitis or benign prostatic hyperplasia, so patients are often diagnosed after failing to treat other diseases or occasionally discovered incidentally while undergoing endoscopic diagnosis and treatment for other diseases. Imaging diagnostic tools such as MRI and PET-CT are used to diagnose invasion, stage, and evaluate distant metastasis. Histopathological diagnosis is made by the increased activity of atypical melanocytes in the epidermis and the separation and necrosis of melanocytes in the dermis. Immunohistochemistry can also be used to detect the expression of melan-A (MART-1), HMB 45, and protein S-100 in atypical melanocyte populations, with single-chain antibodies to protein S-100 reacting with more than 90 % of malignant melanomas [9,10].

The optimal treatment of urethral melanomas is not established, and multiple treatment options are currently used depending on the location of the tumor and clinical stage [11]. The first line of treatment is surgery, but the use of radical surgery did not show any benefit compared to wide local excision with negative surgical margins [12,13]. DiMarco et al. reported that the median time to local recurrence after urethral resection for primary malignant melanoma of the urethra was 6.5 months, and the recurrence rate after 1 year was 60 % [14]. Surgery with lymph node dissection is not recommended for stage A tumors [3,13], however, for patients with groin lymph node metastases an ilioinguinal lymph node dissection is indicated [3]. The prognosis of patients with urethral malignant melanoma from stage B is very poor, with a survival rate of nearly 0 % after 2 years regardless of the treatment method used [7]. Radiotherapy did not show any specific benefit for malignant tumors in the glans and urethra, while chemotherapy and immunotherapy have been used as supportive therapies, but the benefits have not been evaluated due to the small number of patients. Immunotherapy with alpha interferon has been used with reported response rates ranging from 3 % to 23 % [15]. Current strategies for the treatment of advanced and metastatic melanomas are based on targeted therapy with basic molecular mutations and immune signals of tumors. An important consideration regarding the genetics of melanoma is that PD-L1 is highly expressed in mucosal malignant melanoma. The recent study by Mano et al. found 9p24 amplification, which contains the PD-L1 locus [16]. Therefore, PD-1 inhibitors may have a role in the treatment and management of metastatic melanoma [1,17,18].

The case we report in this paper had special features, such as the initial PET/CT scan did not reveal any metastases in the lymph nodes. However, after undergoing bilateral lymphadenectomy by endoscopic surgery, we found lymph node metastasis, possibly due to the rapid progression of the cancer. Despite strict and aggressive adherence to surgery and immunotherapy with Pembrolizumab, the disease seemed intractable and recurred after 5 months, ultimately leading to the patient's death after 18 months. Two recently published case reports using immunotherapy to successfully treat urethral melanoma, an 89-year-old female patient with primary urethral melanoma with lymph node metastasis treated with extensive local resection, EBRT, and adjuvant combination immunotherapy, nivolumab (PD-1 inhibitor) and ipilimumab (CTLA-4), who responded well to treatment after 8 months [19], a 65-year-old man after receiving more than 10 cycles of nivolumab, FDG accumulation was no longer observed on PET-CT, with no recurrence 20 months after nivolumab treatment [20].

Our study limitations are case-report and retrospective.

## 4 Conclusion

To the best of our knowledge, we present a case report on the primary malignant melanoma of the male urethra, which is the first of its kind documented in Vietnam in the English literature. Urethral melanoma with palpable inguinal lymph nodes during an examination is a sign of advanced disease and indicates a poor prognosis.

## Informed consent

Written consent was obtained from the patient's wife to publish this case report. A copy of the written consent is available for review by the Editor-in-Chief of this journal.

## Ethics statement

Not applicable as it is a case report.

## CRediT authorship contribution statement

QN and HN contributed equally as co-first authors and were the main doctors involved in conceiving the original idea, operating on the patients, and designing the study. XB conceived the manuscript, edited it, performed the operation, and wrote the manuscript. TN, VB, and the other authors discussed the results together and contributed to the final manuscript. All authors read and approved the final manuscript.

## Funding

This report involved no sources of funding for any of the authors.

## Publisher's note

All claims expressed in this article are solely those of the authors and do not necessarily represent those of their affiliated organizations, or those of the publisher, the editors, and the reviewers. Any product that may be evaluated in this article, or claim that may be made by its manufacturer, is not guaranteed or endorsed by the publisher.

## Declaration of competing interest

The authors declare that the research was conducted in the absence of any commercial or financial relationships that could be construed as a potential conflict of interest.

## Data Availability

The original contributions presented in the study are included in the article/supplementary material. Further inquiries can be directed to the corresponding author.
